# The Cardiorespiratory fitness of children and adolescents in Tibet at altitudes over 3,500 meters

**DOI:** 10.1371/journal.pone.0256258

**Published:** 2021-08-19

**Authors:** Chaoqun Fan, Ruizhe Sun, Mingjian Nie, Mei Wang, Zhi Yao, Qiang Feng, Wenfeng Xu, Runzi Yuan, Zhongfang Gao, Qiaorui Cheng, Jingjing Wang

**Affiliations:** 1 National Physical Fitness Research Center, China Institute of Sport Science, Dongcheng, Beijing, China; 2 Physical Fitness Research Center, Tibet Institute of Sport Science, Lhasa, Tibet, China; 3 Beijing Sport University, Haidian, Beijing, China; 4 College of Physical Education, Qufu Normal University, Qufu, Shangdong Province, China; Universidade Estadual Paulista Julio de Mesquita Filho - Campus de Bauru, BRAZIL

## Abstract

**Background:**

Cardiorespiratory fitness (CRF) is the core element of health-related physical fitness evaluation. High pressure and low oxygen in Tibet (over 3,500 m above sea level) may negatively impact the residents’ CRF. The 20-m shuttle run test (20mSRT) is the most popular field-based assessment and estimate of CRF in children and adolescents worldwide. However, normative CRF data for the children and adolescents residing in China’s plateau region are unavailable, which prevents comparability among those living at high-altitudes around the world.

**Purpose:**

To measure the CRF of Chinese children and adolescents aged 9–18 years living in Tibet at altitudes exceeding 3,500 m, and to identify correlations between this metric and demographic characteristics (age, sex, and ethnicity). These data were then compared with those generated in the lowland (Shanghai, China) and various global regions.

**Methods:**

20mSRT performance (number of completed laps) and predicted peak oxygen consumption (VO2peak) were used as indicators of CRF. We measured the CRF of 1,717 healthy children and adolescents aged 9–18 years living in Tibet. The CRF data from school-age subjects in Shanghai (2,437 boys and 2,396 girls) and worldwide (1,142,026 students from 50 countries/regions in Africa, Asia, Europe, Latin America, North America, and Oceania) were collated from published papers.

**Results:**

The average CRF of the participants from Tibet was 39.8 mL/kg/min. The male subjects (n = 876; 41.1 ± 4.42 mL/kg/min) had a higher average CRF than their female counterparts (n = 841; 37.8 ± 5.40 mL/kg/min). CRF decreased with age in both sexes at statistical significance (F = 1249.9, *p* for trend 0.05). The indigenous Tibetans (n = 1289; 40.1 ± 3.71 mL/kg/min) had a significant higher average CRF than those of Han descent (n = 394; 38.9 ± 4.70 mL/kg/min) (*p* < 0.05).

**Conclusions:**

Children and adolescents aged 7–18 years residing above 3,500 m in Tibet displayed lower CRF traits compared with their counterparts from the plains area and other high altitude places. CRF varied according to age, sex, and ethnic group. Given the importance of CRF in children and adolescents, effective intervention strategies should be implemented to improve CRF in children and adolescents on the plateau.

## 1. Introduction

Cardiorespiratory fitness (CRF) refers to the integrated ability to deliver oxygen from the atmosphere to the mitochondria to perform physical work. In adults, low CRF is strongly associated with cardiovascular disease and all-cause mortality and morbidity, being independent of adiposity [1–3]. CRF is a more powerful and independent index for evaluating cardiovascular disease and all-cause mortality than traditional risk factors such as hypertension [3], smoking, obesity [4], high cholesterol, and type 2 diabetes. Meanwhile, each increase of 1–2 metabolic equivalents in people with low CRF is accompanied by a 10%-30% reduction in the incidence of cardiovascular disease [5–7]. In 2016, the American Heart Association stated that CRF should be evaluated regularly as the fifth clinical vital sign to predict human health and life span, in addition to respiration, body temperature, pulse, and blood pressure [8].

In children and adolescents, CRF is also an important predictor of cardiovascular disease risk, cancer, and mental health [9, 10]. A high CRF mitigates the negative health impact of being overweight or obese in children and adolescents, referred to as the ‘fat but fit’ phenotype [11]. Therefore, CRF is used as an indicator to comprehensively evaluate the anatomical and physiological coordination ability of children and adolescents participating in physical activities [12, 13]. CRF also tracks subjectively well from childhood to adulthood indicating that monitoring CRF in children provides insight into both current and future population health statuses. Low CRF in children and adolescents increases their risk of cardiovascular metabolic diseases [14, 15], obesity [16], decreased quality of life [17], skeletal dysplasia [18], and mental health [19] in adulthood. In addition to its impact on health and wellness, CRF is an important determinant of youth sports success (e.g., long-distance running, swimming, hockey, basketball, football, rugby) [20].

20mSRT is the most popular field-based assessment and estimate of CRF in children and adolescents worldwide [21, 22]. This exercise test is a powerful, robust tool for investigating and monitoring CRF in large samples, reflected by its good test validity and retest reliability [21, 23]. Furthermore, as a field test method, the 20mSRT’s advantages include low cost, flexible test location (i.e., can be performed indoors or outdoors within a small area), and the ability to test multiple individuals simultaneously with minimal equipment and personnel [24, 25]. To extend the utility of the 20mSRT as a surveillance instrument, it is necessary to specify a universal international standard set based on 20mSRT data collected from different regions and diverse populations. By providing a means with which to compare CRF across geographic areas and time, a universal standard would greatly propel the field of cardiopulmonary function. Olds et al. (2006) reviewed the 20mSRT data from children and adolescents in 37 countries and obtained the average stage and standard score [22]. Tomkinson (2017) used data from 1,142,026 children and adolescents in 50 countries to establish the most comprehensive and up-to-date set of international sex- and age-specific 20mSRT standards for young people aged 9–17 years, which can be applied toward health and fitness screening, profiling and surveillance [26]. However, this standard is not representative of the entire worldwide population, since 20mSRT data has yet to be generated for some regions, living conditions, and demographics [8].

In China, studies have used 20mSRT to measure physical fitness among children and adolescents in the plains region (e.g., Shanghai [27] and Hong Kong [28]). In contrast, few reports have been published for China’s plateau areas. Commonly referred to as the ‘roof of the world’ and ‘the third pole of the globe’, China’s Qinghai-Tibet Plateau is the largest (2.5 million km^2^), highest (> 4,000 m), and most densely populated (> 12 million) plateau in the world [29]. Tibetans are the main indigenous people residing on the Qinghai-Tibet Plateau. They settled the Qinghai-Tibet Plateau as early as 30,000 years ago and began to physiologically adapt to its low-oxygen environment [30]. Many lowland people (mainly of Han descent) have since migrated to the plateau in response to the Chinese western development strategy and “Go West” campaign. The Sixth National Population Census of China (2010) showed that the indigenous Tibetan and Han subpopulations account for 90.48% and 8.17% of the total population of Tibet, respectively; as such, these are the two main ethnic groups in the Qinghai-Tibet Plateau [31]. The conditions characteristic of this area (e.g., low oxygen, low temperature, strong ultraviolet radiation, and extreme climate fluctuations) render the regional environments of Tibet and inland China very different. Tibet’s extreme living environment is a great challenge to human survival and reproduction. Living at altitudes over 3,000 m impacts human health [32, 33]. Chronic mountain sickness, congenital heart disease, and osteoporosis have higher incidence rates and regional characteristics in Tibet than those in the plain [34, 35].

Several rounds of China’s National Physical Fitness Surveillance have shown that the CRF of children and adolescents in Tibet was lower than their peers in neighboring provinces [36]. However, this surveillance program uses the 800/1,000-m run test to evaluate CRF. Unfortunately, this assessment has a lower validity index and is less frequently used worldwide than the 20mSRT; thereby limiting cross-regional comparison analyses. Therefore, this study quantified CRF using the 20mSRT among children and adolescents in Tibet and analyzed the resulting dataset for correlations with ethnicity, sex, and age. Additionally, this study compared the CRF of children and adolescents in Tibet (high-altitude area) with those of their counterparts living in the low-altitude regions of China and global averages. The findings of this study provide high-altitude and Tibetan data to incorporate into the global standard for children’s and adolescents’ CRF with 20mSRT.

## 2. Materials and methods

### 2.1. Design and participants

Given Tibet’s unique geographical environment and low population density, the schools are concentratively distributed. Over half of the high schools and two-thirds of junior high schools are located in the Chengguan District (one out of a total of eight districts in the city) of the city of Lhasa. Therefore, three primary schools, two junior high schools, and two senior high schools were randomly selected in the Chengguan District of Lhasa 3500m).

Data were collected from July to September 2019. As a part of the “Investigation on Physical Fitness and Health Behaviors of Tibetan School-Age Children and Adolescents (IPFHB-TSCA)” conducted by the Tibet Institute of Sport Science (TISS) and the China Institute of Sport Science (CISS), this study was approved by the CISS Ethics Committee (number: CISSRB-2016006). Written informed consent was provided by the students and their guardians prior to the commencement of this study and all students’ names were digitally coded to ensure confidentiality.

More than 2,000 students aged 9–18 years (4th - 12th grade) were recruited from seven schools. From each grade, approximately 180 students were randomly selected at a male to female ratio of 1:1 and a Tibetan to Han ratio of 4:1. According to educational organization statistics of Tibet, the ethnicity ratio of children and adolescents in Tibet is about 8:1. However, considering the large age span between the study subjects (9 to 18 years), the feasibility of recruiting Han students, and the sample size from each ethnicity required to fulfill the statistical requirement, we set the ethnic sampling ratio at 4:1 in this study.

The eligibility criteria were to: 1) be able to carry out normal physical activities; 2) understand and be willing to complete all of the test requirements; 3) have no known disease, injury, or other physiological problem; and 4) have no specialized sports training experience. Students were excluded if they answered “yes” to any question on the Physical Activity Preparation Questionnaire (PAR-Q) [37], which indicated that performing the 20mSRT could potentially put them at risk.

### 2.2. Measures

In the IPFHB-TSCA project, students were assessed using a lifestyle questionnaire, health-related physical fitness test, and the 20mSRT. Only demographic characteristics, BMI, and 20mSRT data are reported in the current sub-study.

#### 2.2.1. Demographic characteristics

Demographic data were obtained through a questionnaire and included sex, ethnicity, birth date, test date.

#### 2.2.2. Height, weight, and Body Mass Index (BMI)

Height and weight were measured using a validated electronic height and weight scale (Jianmin Brand, Beijing, China) according to a standard protocol. Height was accurate to 0.1 cm, and weight was accurate to 0.1 kg. BMI was calculated as the weight in kilograms divided by the square of the height in meters.

#### 2.2.3. Cardiorespiratory fitness in children and adolescents in Tibet

CRF was assessed using 20mSRT to estimate peak oxygen uptake (VO_2peak_). The 20mSRT was carried out on a flat outdoor field or runway (outdoor). First, the tester explained the experimental procedure and played the test music to familiarize the students with the rhythm. Then, the students did warm-up exercises (e.g., joint movement, muscle stretching) for 5–10 minutes. During the initial stage of formal testing, the tester let the students run to the finish line with the rhythm to ensure that they could follow the speed. The speed started at 8.0 km/h and increased to 9.0 km/h at the second level, followed by 0.5 km/h-increases each subsequent minute thereafter. The running speed was strictly controlled by the audio rhythm to ensure that the students’ physical load remained the same. When a student was too fast or too slow, the tester instructed them to adjust their speed accordingly. The test was terminated when participants stopped running due to fatigue, or when they failed to follow the rhythm twice before reaching the finish line. The laps were considered as completed only when both of the student’s feet stepped over the finish line. Given that this test is quite exhausting and was carried out in a high-altitude, low-oxygen environment, the termination conditions were strictly monitored during the test to prevent the occurrence of any adverse events.

The number of laps completed by all participants was recorded and the VO_2peak_ of Tibetan children and adolescents was estimated by using Leger’s equation [38]. This approach has a moderate-to-high criterion-related validity (r _p_ = 0.78, 95% confidence interval [CI] [0.72–0.85]) for estimating VO_2peak_ [39] and a test-retest reliability coefficient of 0.89 for children; therefore, it is frequently used to estimate VO_2peak_ worldwide [26, 40, 41]

MAS = 8 + 0.5×Stage

VO_2peak_ = 31.025 + 3.238*MAS—3.248*age + 0.1536*MAS*age,

Sixty-eight adolescents aged 11–15 years were selected to test the validity of the 20mSRT. The subjects were required to complete the cardiopulmonary exercise test and the 20mSRT with an interval of one week. The VO_2peak_ was estimated based on the laps from the 20mSRT using the Leger formula. VO_2peak_ was measured in a standardized and laboratory-based incremental test to exhaustion. The students took part in the gradual-loading-exercise test on the bicycle ergometer with initial load at 25 w by 25 w increase every two minutes and the speed at 60 rotations per minute. Brachial artery blood pressure was measured and recorded every two minutes and cardiac rhythm was monitored using a 12-lead ECG (Cardio Perfect, New York, USA) during the exercise test. Oxygen uptake, carbon dioxide output, and minute expired ventilation were measured breath-by-breath using a calibrated open-circuit metabolic measurement system (Cortex Metalyzer Ⅱ, Berlin, Germany). Before the test, the metabolic measurement system was corrected strictly for gas composition and pressure in plateau environment. The correlation coefficient between the 20mSRT-based VO_2peak_ estimation and the VO_2max_ determined using the cardiopulmonary exercise test was 0.68 (0.71:0.63 boys to girls; 0.73:0.61 Tibetan to Han), which supported the validity of using the 20mSRT to quantify the CRF on the Tibet Plateau.

### 2.3. Cardiorespiratory fitness in regional and international populations

The CRF of children and adolescents living in Tibet was determined using the aforementioned 20mSRT-based method. The CRF data from 4,833 children and adolescents aged 7–17 years (2,437 boys and 2,396 girls) living in Shanghai were published in 2018 [27]. Similarly, the CRF data describing an international population (1,142,026 children and adolescents from 50 distinct countries/regions in Africa, Asia, Europe, Latin America, North America, and Oceania) were published in 2017 [26].

### 2.4. Statistical analysis

The results are presented as numbers with relative percentages for categorical data and as means with standard deviation (SD), P10, P50, and P90 for continuous data. The Shapiro-Wilk test was performed to ensure the data were normally distributed. One-way ANOVA and trend test were used to identify differences in 20mSRT performance and VO_2peak_ according to age; while an independent sample t-test was applied to explore differences according to ethnicity and sex. An abstract independent sample t-test was used to identify differences in 20mSRT performance and VO_2peak_ between students from Shanghai and Tibet with respect to age, ethnicity, and sex. Statistical significance was set at p < 0.05 and all analyses were conducted using the statistical software SPSS version 24.0.

## 3. Results

### 3.1. Demographic characteristics

The study participants’ demographic characteristics are presented in Table 1. Of the 1,974 school-aged children and adolescents who participated in the survey, 1,717 samples (876 boys and 841 girls) provided valid data. Participants’ mean age was 14.0 ± 2.7 years and the population contained 1,289 (76.9%) Tibetan and 394 (23.1%) Han students.

**Table 1:** Description of the study sample.

Study Variables Variables	Sample N (%)	Hight(m) M(SD) M(SD)	Weight(kg) M(SD)	BMI(kg/m^2^) M(SD) M(SD)
**Overall**	1717	155.7(12.5)	46.4(12.3)	18.8(2.8)
**Age**				
**9**	126(7.33%)	135.5(6.5)	30.1(6.3)	16.3(2.2)
**10**	175(10.1%)	140.5(7.2)	32.3(6.1)	16.3(2.2)
**11**	166(10.0%)	145.5(7.6)	37.2(7.9)	17.4(2.6)
**12**	173(10.0%)	152.3(8.5)	42.1(8.9)	18.0(2.7)
**13**	188(10.8%)	158.4(8.2)	47.7(9.6)	18.9(2.9)
**14**	200(10.8%)	162.2(7.2)	50.4(7.9)	19.1(2.3)
**15**	201(11.6%)	163.0(7.1)	53.8(8.9)	20.2(2.9)
**16**	205(11.8%)	163.9(7.6)	54.3(8.3)	20.2(2.7)
**17**	175(10.1%)	164.5(7.9)	55.6(9.2)	20.5(2.9)
**18**	108(6.2%)	164.8(8.6)	55.6(9.6)	20.7(2.8)
**Gender**				
**Boys**	876(51.0%)	158.5(13.9)	48.3(13.5)	18.7(3.1)
**Girls**	841(49.0%)	152.3(9.7)	44.5(10.5) *	18.8(3.0)*
**Ethnicity**				
**Tibetan**	1289(76.9%)	155.8(12.2)	46.5(11.8)	18.9(2.7)
**Han**	394(23.1%)	155.2(13.3)	45.8(13.6)	18.6(3.3)^&^

N = sample size of children and adolescents in the corresponding group; M = Mean; SD = Standard Deviation

BMI = Body Mass Index.

*P<0.05 vs. boy group

^&^*P*<0.05 vs. Tibetan group.

### 3.2. Differences in CRF according to age and sex

Table 2 presents the sample’s 20mSRT performance, including the number of completed shuttle laps and predicted VO_2peak_ (mL/kg/min). The number of completed laps ranged from 21.0 to 35.4 shuttles, and both sexes exhibited a statistically significant growth trend with age (F = 1249.9, p for trend <0.05). However, predicted VO2_peak_ (mL/kg/min) decreased with age, ranging from 45.6 mL/kg/min at 9 years old to 33.3mL/kg/min at 18 years old (F = 4285.8, *p* for trend <0.05). The laps and predicted VO_2peak_ of the boys were significantly higher than those of the girls except among 11-year-olds (*p* < 0.05).

**Table 2:** 20m shuttle run test (20mSRT) performance (number of laps) and predicted VO_2peak_ (mL/kg/min) of boys and girls aged 9–18 years in Tibet.

Age	N	N1	N2	laps M(SD)			VO_2peak_ M(SD)		
				boys	girls	overall	boys	girls	overall
**Overall**	1717	876	841	36.13(16.03)	22.04(7.40) *	35.4(18.3)	41.07(4.42)	37.76(5.40) *	39.8(5.3)
**9**	126	61	65	22.70(8.06)	19.48(5.52)*	21.0(7.0)	46.27 (2.37)	45.06 (1.85) *	45.6(2.2)
**10**	175	87	88	24.35(9.26)	20.07(5.50)*	22.2(7.8)	44.43 (2.87)	43.22 (1.73) *	43.8(2.4)
**11**	166	77	89	25.26(11.52)	22.29(7.06)	23.5(9.4)	42.84 (3.01)	42.33 (2.37)	42.5(2.6)
**12**	173	86	87	29.32(10.44)	22.35(7.23) *	25.7(9.6)	42.42 (2.99)	40.53 (2.32) *	41.4(2.8)
**13**	188	94	94	33.98(13.44)	23.90(8.31) *	28.9(12.2)	42.12 (3.94)	39.10 (2.83) *	40.6(3.7)
**14**	200	105	95	40.07(14.04)	24.33(8.32) *	32.6(14.0)	42.07 (3.88)	37.54 (2.85) *	39.8(4.1)
**15**	201	110	91	41.65(15.00)	22.14(7.57) *	33.1(15.6)	40.96 (4.56)	35.01 (2.83) *	38.3(4.8)
**16**	205	108	97	42.84(15.76)	21.64(8.21) *	34.8(16.5)	39.42 (4.64)	33.16 (3.10) *	36.4(5.0)
**17**	175	96	79	47.63(16.22) #	21.03 (7.08) *	35.0(18.5)	39.26 (4.70)	31.06 (3.04) *	35.3(5.7)
**18**	108	52	56	50.12(15.54)	21.80(5.48) *	35.0(18.5)	37.76(4.32)	29.65(2.55) *	33.3(5.3)
*p* **for trend**				*p* < 0.05	*p* < 0.05	*p* < 0.05	*p* < 0.05	*p* < 0.05	*p* < 0.05

N = Sample size of children and adolescents in Tibet; N1 = Sample size of boys in Tibet; N2 = Sample size of girls in Tibet; M = Mean; SD = Standard Deviation.

* *p* < 0.05 vs. the boys in the same age group.

### 3.3. Differences in CRF according to ethnicity

Table 3 shows the 20mSRT-based CRF differences among 9- to 18-year-old children and adolescents residing in Tibet according to ethnicity. As a whole, the Tibetan subjects completed more laps than those of Han descent (30.4 shuttles vs. 25.3 shuttles, *p* < 0.05). The statistical significance comes mainly from the difference between the laps of Tibetan- and Han-nationality boys and girls aged 11–13 and 15 years. The predicted VO_2peak_ of Tibetan children and adolescents was also higher than that of Han children and adolescents (40.1 mL/kg/min vs. 38.9 mL/kg/min, *p* < 0.05), among which there were statistically significant differences in predicted VO_2peak_ between Tibetan and Han boys aged 11–14 and 17 years, and between Tibetan and Han girls aged 10, 12–13, and 17–18 years.

**Table 3:** 20m shuttle run test (20mSRT) performance (number of laps) and predicted VO_2peak_ (mL/kg/min) of Tibetan and Han students aged 9–18 years in Tibet.

Sex	Age	N1	N2	Laps M(SD)		VO_2peak_ M(SD)	
				Tibetan	Han	Tibetan	Han
**overall**	**14.3**	**1289**	**394**	**30.4(15.1)**	**25.4(10.9)** ^&^	**40.1(5.4)**	**38.9(4.7)** ^&^
	**Overall**	661	209	37.85(16.66)	30.33(12.16) ^&^	42.15(4.50)	40.37(3.71) ^&^
	**9**	46	15	23.35(8.57)	20.57(5.89)	46.55 (2.50)	45.38 (1.67)
	**10**	67	20	24.65(9.10)	23.40(9.95)	44.62 (2.72)	43.80 (3.30)
	**11**	60	17	27.18(12.01)	23.47(6.05) ^&^	43.43 (2.99)	40.79 (2.05) ^&^
**Boys**	**12**	65	21	30.67(10.94)	24.50(6.74) ^&^	42.75 (3.13)	41.24 (2.12) ^&^
	**13**	77	17	36.50(13.52)	24.60(4.94) ^&^	42.80 (4.00)	39.16 (1.70) ^&^
	**14**	79	26	42.31(14.18)	33.16(11.26) ^&^	42.62 (3.90)	40.42 (3.38) ^&^
	**15**	75	34	44.53(16.25)	35.38(9.66) ^&^	41.68 (5.00)	39.40 (3.05)
	**16**	80	26	44.90(17.26)	40.54(9.92)	39.58 (5.02)	39.16 (3.36)
	**17**	75	18	49.76(16.25)	40.00(11.16) ^&^	39.92 (4.61)	36.67 (3.75) ^&^
	**18**	37	15	52.26(15.51)	44.78(15.44)	38.94(4.20)	36.94(4.96)
	**Overall**	628	185	22.67(7.72)	20.03(5.64) ^&^	37.97(5.42)	37.36(5.23)
	**9**	50	15	19.37(6.05)	19.87(3.42)	45.08 (2.00)	45.01 (1.31)
	**10**	65	21	20.59(5.70)	18.43(4.87)	43.46 (1.77)	42.57 (1.45) ^&^
**Girls**	**11**	70	19	23.48(6.93)	19.17(6.86) ^&^	42.58 (2.22)	41.36 (2.71)
	**12**	58	22	22.46(7.15)	19.50(4.55) ^&^	40.76 (2.50)	39.81 (1.41) ^&^
	**13**	71	19	24.63(8.67)	21.00(7.03) ^&^	39.35 (2.86)	38.16 (2.56) ^&^
	**14**	70	21	24.87(8.69)	21.71(6.51)	37.77 (2.98)	36.74 (2.22)
	**15**	68	20	22.68(7.62)	20.06(6.12) ^&^	35.34 (2.84)	34.02 (2.63)
	**16**	74	21	22.11(8.84)	20.57(5.49)	33.28 (3.32)	33.02 (2.25)
	**17**	59	17	21.19(7.87)	20.13 (6.87)	31.14 (3.09)	30.68 (2.66) ^&^
	**18**	43	10	22.60(5.69)	18.40(2.95)	30.03(2.60)	27.82(1.65) ^&^

N1 = Sample size of Tibetan students in Tibet; N2 = Sample size of Han students in Tibet; M = Mean; SD = Standard Deviation.

^&^*P* < 0.05 vs. Tibetans in the same age group.

### 3.4. Difference of in CRF between children and adolescents according to region

**Fig 1:**
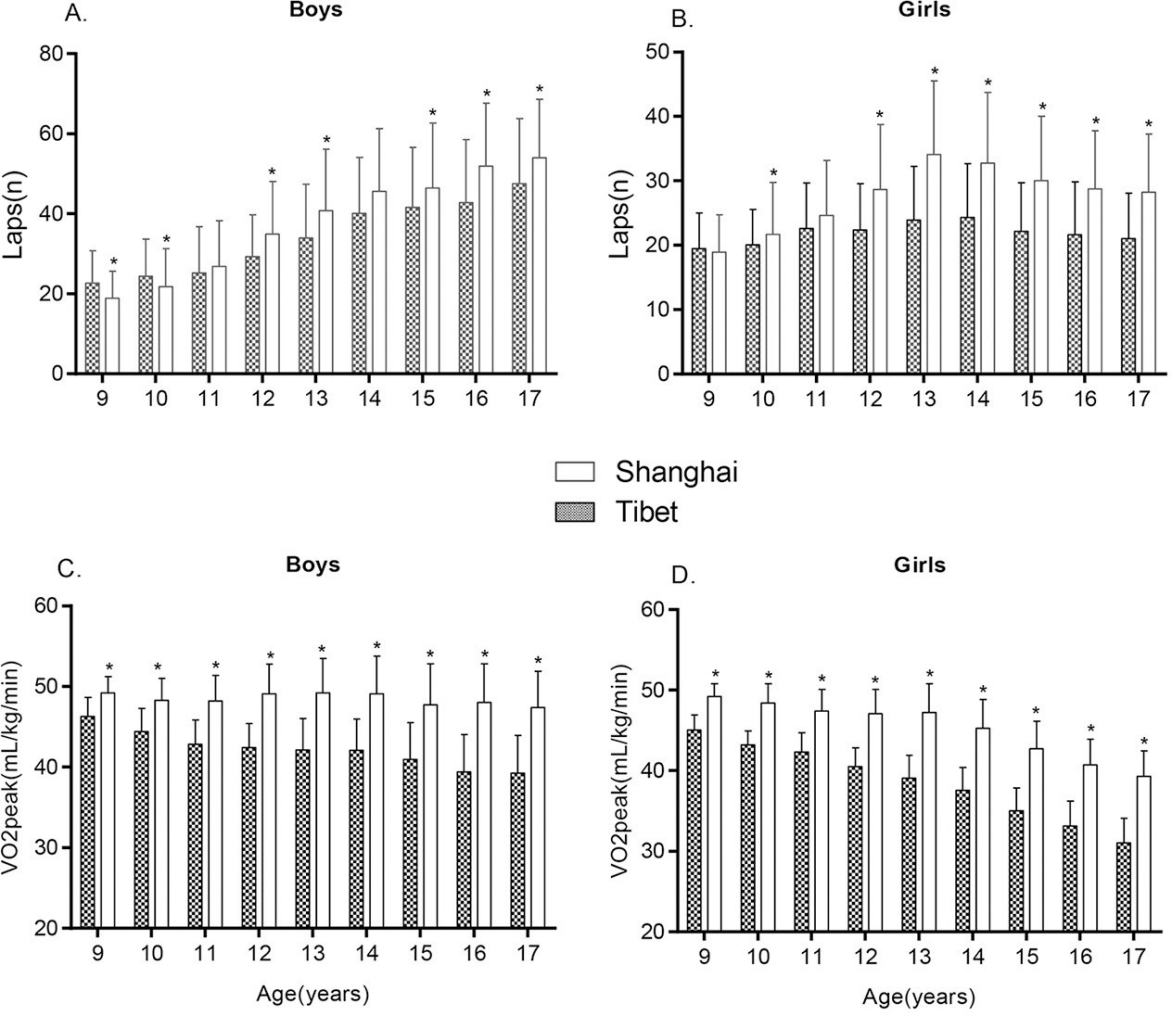
Laps(n) and VO_2_peak(mL/kg/min) in 20mSRT of students aged 9–17 years in Tibet and Shanghai. (A) 20mSRT performance (laps) in boys; (B): 20mSRT performance (laps) in girls; (C): Predicted VO_2peak_ in boys”; (D): Predicted VO_2peak_ in girls; “20mSRT” refers to the 20m shuttle run test; “Tibet” refers to children and adolescents aged 9–17 years residing in Tibet; “Shanghai” refers to children and adolescents aged 9–17 years from the Shanghai Province. All values are presented as means. *p < 0.05 vs. Tibet group.

Fig 1 presents the differences in 20mSRT laps completed by students in Tibet and Shanghai. Generally, the mean laps of boys in both Tibet and Shanghai showed an increasing increased with age. The laps of Tibetan girls grew gradually from 9 to 14 years old and then decreased gently, similar to the trend observed in girls residing in Shanghai. The laps completed by students in Tibet were less than those in Shanghai in all of the age groups except for 11- and 14-year-old boys and 9- and 11-year-old girls.

Fig 1 also lists the differences in predicted VO_2peak_ between students in Tibet and Shanghai aged 9–17 years. Among the boys and girls from both regions, VO_2peak_ decreased gradually with age. The VO_2peak_ of boys and girls in Tibet were significantly lower than their low-altitude counterparts in Shanghai.

### 3.5. Difference in CRF between children and adolescents in Tibet and worldwide

Table 4 shows the P10, P50, and P90 values for 20mSRT performance (laps) of children and adolescents aged 7–18 years in Tibet and worldwide. Together, these data indicate that the boys and girls in Tibet performed poorly compared with those from other regions. The absolute values of P10, P50, and P90 for laps completed by boys in Tibet and worldwide increased with age. In contrast, the P10, P50, and P90 values for the laps completed by girls in Tibet improved from 9 to 14 years old but decreased from 14 to 17 years old.

**Table 4:** 20m shuttle run test (20mSRT) performance (number of laps) and predicted VO_2_peak (mL/kg/min) centiles by age and sex in Tibet and the world’s students aged 9–17 years.

Sex	Age	laps						VO_2_peak					
		Tibet			the world			Tibet			the world		
		P10	P50	P90	P10	P50	P90	P10	P50	P90	P10	P50	P90
	**9**	14	22	35	12	19	29	43	45	48	43	48	53
	**10**	15	23	39	10	20	29	40	44	48	41	47	53
	**11**	16	26	42	12	21	29	38	41	45	40	46	52
**Boys**	**12**	19	30	46	15	25	29	39	41	43	38	45	52
	**13**	16	38	53	17	26	31	37	38	41	37	45	53
	**14**	21	43	57	18	35	43	35	41	45	36	45	53
	**15**	19	48	63	23	36	48	35	40	43	35	44	53
	**16**	19	46	68	29	38	56	35	38	45	33	43	53
	**17**	26	52	69	23	39	58	31	37	42	32	43	53
	**9**	13	18	28	15	18	25	44	44	47	43	47	51
	**10**	13	21	28	12	18	25	40	43	45	40	45	50
**Girls**	**11**	14	24	33	10	18	27	38	42	44	38	44	49
	**12**	14	24	36	13	20	26	38	40	42	36	42	48
	**13**	15	24	38	12	21	32	35	38	41	34	41	47
	**14**	14	25	36	11	22	32	34	36	41	32	39	46
	**15**	14	23	34	13	20	29	31	34	38	31	37	44
	**16**	11	21	35	13	20	28	30	33	36	29	36	43
	**17**	12	20	30	13	20	32	27	31	34	27	34	42

P = Percentile.

Table 4 also lists the P10, P50, and P90 values for the predicted VO2peak of children and adolescents aged 7–18 years in Tibet and worldwide. For boys and girls in Tibet, the absolute values of the P10, P50, and P90 predicted VO2peak were lower than their international counterparts. The P10, P50, and P90 predicted VO2peak in both sexes tended to decrease as age increased, though this trend was subtle in boys.

## 4. Discussion

This study assessed CRF among 1,717 children and youth in Tibet aged 9–18 years. Age-, sex-, and ethnicity-based differences were detected in the CRF of this population. While sports performance tends to increase with age, the participants’ CRF decreased from 45.6 mL/kg/min at 9 years old to 33.3 mL/kg/min at 18 years old. At most ages, the boys had higher CRF values than the girls, and Tibetan students performed better than Han students. Compared with children and young adults in Shanghai (the plains region, close to sea level) and worldwide (from 50 countries), the children and adolescents in Tibet had lower CRF.

Several studies have explored the relationship between age and CRF in children and young adults. Olds et al. integrated the original data from 109 studies representing 37 countries from 1981 to 2003 (n = 418,026) and found that CRF decreases as age increases [22]. Tomkinson’s study of the percentile curve of 117 items (n = 1,142,026) collected in 50 countries from 1945 to 2015 further supports this trend [26]. In line with these studies, this present cross-sectional study found that CRF among boys and girls aged 9–18 years in Tibet decreased gradually with age. This was attributed mainly to differences in physical activity (PA) levels [42, 43], changes in cardiac output, and increases in bodyweight [44]. As they age, students in China face more academic pressure, which may result in more time spent sedentary while studying and less time spent exercising. Decrease PA directly leads to a reduction in CRF [45]. Their oxygen supply capacity, which causes their maximum oxygen uptake capacity to decrease. In the later stages of puberty, the bodyweight of both boys and girls rises sharply with age, though their oxygen uptake capacity does not increase accordingly. Thus, VO2peak as a unit per kilogram of bodyweight presents a decreasing trend [46].

The present study revealed a sex-based difference in the CRF of students in Tibet. The VO2peak of boys was significantly higher than that of girls. In addition to the fact that boys maintain a high altitude [47–49], it may be related to sex hormones. Sex-based differences cause significant changes in body fat tissue and lean bodyweight composition, and differences in hemoglobin content between pubertal boys and girls may be a critical component of the sex-based differences in CRF [50]. However, girls’ VO2peak being lower than boys’ is not related to higher health risk but, rather, to the physiological function of girls. According to the FitnessGram standard (USA), the VO_2max_ of girls is relatively lower than that of boys due to physiological characteristics [51]. Only when the VO_2max_ of children and adolescents dips below a certain level are health risks elevated in later adulthood [52].

The present study also found that Tibetan students had greater cardiopulmonary endurance than Han students residing in Tibet, despite living in the same environment. These findings are hard to put into context given the scarcity of studies exploring CRF in different ethnicities. Through Berntsen’s study from Lhasa in Tibet [53], 40 children aged 9–10 years-old born in Lhasa participated in a direct test of cardiopulmonary endurance using a cycle ergometer. The results showed that the aerobic capacity of boys was significantly higher than that of girls, but there was no significant difference between Tibetan and Han students. Presumably, any ethnic differences between groups at the same altitude may be partially explained by adaptation, length of migration, PA level, and lifestyle and cultural differences in the plateau. The unique conditions of the plateau environment include but are not limited to low pressure, low oxygen level, low humidity, cold weather, strong solar infrared and ultraviolet radiation. These characteristics affect the human body and lifestyle and are not conducive to human survival [54]. As an indigenous group, Tibetans have developed physiological adaptations to their high-altitude, hypoxic environment [55]. For example, in terms of morphology, Tibetan aborigines have obvious sternum depth in boys and sternum width in girls [56, 57]. Physiologically, the typical adaptation characteristics of those who live on the Tibetan Plateau include a high ventilation rate, increased blood oxygen saturation, low pulmonary artery pressure, and decreased hemoglobin concentration [55, 58]. Not until the 1970s did Han individuals begin to move into Tibet on a large scale [59], at which point they began to gradually adapt to the plateau environment. However, their adaption to this specialized environment may not be as developed as that of the indigenous ethnicity that has lived there for generations. Tibetan children and adolescents are more active than their Han counterparts. The sports ability of Tibetan aboriginal youth is also greater than that of Han immigrants [59, 60]. Moreover, three genes related to natural selection in Tibetans—EPAS1, EGLN1, and PPARa—are associated with their relatively low hemoglobin concentration [61]. In the daily life of Tibetans, there are many religious activities of moderate to high intensity, such as turning galleries, climbing mountains, prayer wheels, and the long kowtow [62, 63]. These daily activities—in which those of Han descent are seldom involved—may contribute to the high CRF levels of Tibetan aborigines. Altitude training is a practice in which athletes from the plains regions move onto the plateau to increase red blood cells counts through hypoxic stimulation, improve their blood’s oxygen-carrying capacity, increase their tidal volume and alveolar blood flow, enhance their pulmonary ventilation to blood flow ratio, improve pulmonary diffusion, redistribute their peripheral blood flow, reduce their heart rates, elevate their heart stroke volume, improve the utilization rate of oxygen, and enhance their endurance, which collectively improve athletic performance [64–66]. The advantages conferred by altitude training may also partially underlie the higher CRF in Tibetan aborigines compared with the Hans, who migrated relatively recently.

Compared with their peers living in the plains region (Shanghai) of China and worldwide, children and adolescents in Tibet had a lower average CRF. The two studies published on the plateau area report inconsistent findings. Smith et al. [67] showed aerobic endurance increases at high altitude (600–1,000 m); however, their altitude range is much lower than our study. In contrast, Chen [68] found that, at altitudes (3,417–4,300 m) similar to the present study, maximum oxygen uptake was inversely correlated with altitude in children and adolescents. In the current study, children and adolescents in Tibet had a lower average CRF than their peers living at lower altitudes in Shanghai, China and compared with integrated data from 50 countries throughout the world. This difference can be attributed to the altitude, economic and educational level, developmental state, and lifestyle. At sea level (Shanghai), the standard barometric pressure is 760 mmHg, while the oxygen partial pressure is 160 mmHg in the atmosphere and 100 mmHg in arteries. Meanwhile, at an altitude of 3,500 m (Lhasa, Tibet), the standard barometric pressure drops to 459 mmHg, while the oxygen partial pressure in the atmosphere and arterial oxygen decreases accordingly. A 40% reduction in arterial oxygen partial pressure decreases arterial oxygen saturation (SaO2 = 89%), although the individuals living at this altitude have developed molecular adaptations, for example, in erythropoietin regulation [69–71]. Nipa’ study explored significantly lower red blood cells count, hematocrit, hemoglobin concentration, mean corpuscular hemoglobin, mean corpuscular hemoglobin concentration and higher mean corpuscular volume in long-term Tibetan residents living at low altitude compared to their high altitude counterparts, which is likely due to the outcome of hematological adaptation to a relatively hyperoxic environment in low altitude areas [72]. However, the quantified effect of the differences of the abovementioned blood indexes on CRF in students at different altitudes is not clear, which needs further exploration. Relatively, it is clear that with the increase of altitude, the arterial oxygen partial pressure decreases, and the working ability and exercise ability of residents decrease [69, 73]. A previous study also found that VO_2max_ and altitude have a negative, linear relationship; they describe a 6–7% decrease associated with each 1,000-m increase in altitude [74]. Altitude is likely the main factor underlying the CRF reduction among children and adolescents in Tibet. Furthermore, although the children and adolescents in this study were recruited from the most developed city (Lhasa) in Tibet, their economic level is among the lowest of China’s 31 provinces according to the official GDP ranking in 2020. Conversely, as a megacity with a highly developed economy and culture, Shanghai has a top-ranking GDP. Furthermore, Shanghai’s being at the forefront of China’s sports industry could bolster children’s physical exercise behavior through support from their families, schools, and society in general. Taken together, these factors all likely contribute to a higher PA level in students in Shanghai, which could improve CRF [48]. Additionally, the growth rate of children living in the highest-altitude area (4,040 m) is around two years behind their peers residing at an altitude of 2,261 m [75], which may be another reason why the CRF of children and adolescents at high altitude was lower than that of their peers. Moreover, a comparative study showed that the prevalence of overweight and obesity in children and adolescents aged 7–18 years in Tibet increased annually over the past 20 years [76], due to changes in dietary habits. Due to the high price of fruits and vegetables in the plateau area, the intake of these healthy foods is lower than the recommended amount. Finally, the cold, anoxic environment results in less time suitable for exercise, as engaging high-intensity exercise outdoors for a long time is difficult in such an environment. All of these factors may contribute to adiposity and affect the CRF level. Unlike the gradually increasing trend among girls internationally, the laps completed by girls in Tibet decreased from 14 to 17 years old. This trend was consistent with that of Shanghai girls. More research is needed to identify the reasons underlying this phenomenon.

It is surprising that while the VO2peak predicted by 20mSRT decreased across all ages in the present study, 20mSRT performance (laps) did not follow this trend. The boys aged 9–18 years and girls aged 9–14 years had CRF values that increased with age, consistent with Tomkinson’s study [26]. However, a slowly declining trend was observed in girls older than 14 years, and also in students from Shanghai. The 20mSRT is also known as progressive aerobic cardiovascular endurance run (PACER) [77]. With the increase in age, long-distance running becomes more economical, meaning that older children run farther at the same running speed or run the same distance faster [78]. There are existing studies that directly evaluate aerobic endurance using the laps of 20mSRT rather than calculated maximum oxygen uptake. However, the discrepancy in the trend between completed laps and peak oxygen uptake with the 20mSRT should be addressed. Evaluating CRF by 20mSRT laps alone may overestimate the CRF level in late adolescence because it does not take age, weight, and body composition into account. In adults, laps seem to be a more accurate estimator of CRF, but the resulting performance score should be combined with other indices to estimate CRF in children [79]. Nevertheless, examiners should be aware of the fact that performance scores derived from the 20mSRT alone are only estimations not direct measures of CRF.

Investigating the CRF among children and adolescents living at 3,500 m in Tibet should help to establish a Chinese evaluation standard for CRF. Furthermore, the regional and international comparisons of CRF in children and adolescents supplements data from other plateau regions in international studies on CRF. These findings provide the basis for implementing an effective CRF evaluation and promotion strategy among Tibetan children and adolescents.

The present study has some limitations. First, the study was conducted only in Lhasa and used quota rather than random sampling. However, considering the specialized geographical environment of Tibet, the geographical distribution of schools at all levels, and the difficulty of sampling sufficient Han students, the samples are still representative. Second, the data were obtained from June to September, which is the most pleasant season in Tibet. Therefore, the results likely reflect the highest CRF levels among children and adolescents living in Tibet throughout the year. Third, CRF was estimated by an equation based on 20mSRT rather than a direct measure. The different prediction models could generate different values, although Leger’s equation has a wide acceptance and application. Forth, the findings from this cross-sectional study must be verified by longitudinal or cohort studies. The trend of change in CRF with age must be verified by longitudinal or cohort studies. Fifthly, in comparison of CRF, we did not concern the impact of hematological adaptation on the VO_2max_ of students in sea-level and mountainous regions. It is still the need for more in-depth and meticulous research. Lastly, the present study did not analyze children’s and adolescents’ lifestyles, such as PA, sleep, and dietary behaviors. Further cohort studies and lifestyle surveys should be conducted in children and adolescents in Tibet to examine the influence of lifestyle factors on CRF promotion.

## 5. Conclusions

The overall CRF of children and adolescents aged 7–18 years residing above 3,500 m in Tibet was lower than their counterparts from the plains area and the global average. CRF differed according to age, sex, and ethnicity. Boys had higher CRF than girls. With the increase of age, the CRF in both sexes decreased. Native Tibetans showed better CRF than Hans. Children and adolescents also displayed lower CRF traits compared with their counterparts from the plains area and other high altitude places. These findings can serve as reference and data support for the evaluation of the CRF in children and adolescents in Tibet and other high-altitude areas. Given the benefits of CRF in children and adolescents, effective intervention strategies should be implemented to improve CRF.

## Supporting information

S1 Fig Differences in 20m shuttle run test performance (number of laps) and predicted VO_2_peak (mL/kg/min) between children and adolescents aged 9–17 years in Tibet and Shanghai. (DOCX)
