# Correction: The Cardiorespiratory fitness of children and adolescents in Tibet at altitudes over 3,500 meters

**DOI:** 10.1371/journal.pone.0296556

**Published:** 2023-12-28

**Authors:** Chaoqun Fan, Ruizhe Sun, Mingjian Nie, Mei Wang, Zhi Yao, Qiang Feng, Wenfeng Xu, Runzi Yuan, Zhongfang Gao, Qiaorui Cheng, Jingjing Wang

There are errors in the Funding section. The correct Funding statement is: This research was funded by the Fundamental Research Funds for the China Institute of Sport Science (grant no.19-32 and 20–14) and the Funds of Department of Science and Education, General Administration of Sport of China (grant number 2015B121,2015B120).
