# Supplementary material for: The Cardiorespiratory fitness of children and adolescents in Tibet at altitudes over 3,500 meters
Source: PLoS One. 2021 Aug 19;16(8):e0256258. doi: 10.1371/journal.pone.0256258 (PMC8375997; doi:10.1371/journal.pone.0256258)
Supplement: S1 Fig — (DOCX) [file pone.0256258.s001.docx]

**S1_Fig**

**Differences in 20m shuttle run test performance (number of laps) and predicted VO_2_peak (mL/kg/min) between children and adolescents aged 9-17 years in Tibet and Shanghai**

| **Sex** | **Age** | **N1** | **N2** | **Laps M(SD)** | | **VO_2_peak M(SD)** | |
| --- | --- | --- | --- | --- | --- | --- | --- |
|  |  |  |  | **Tibet** | **Shanghai** | **Tibet** | **Shanghai** |
| **Boys** | **9** | 60 | 189 | 22.70(8.06) | 18.91(6.75)^&^ | 46.27 (2.37) | 49.2 (2.05) ^&^ |
|  | **10** | 85 | 185 | 24.35(9.26) | 21.77(9.51) ^&^ | 44.43 (2.87) | 48.3 (2.71) ^&^ |
|  | **11** | 77 | 175 | 25.26(11.52) | 26.81(11.51) | 42.84 (3.01) | 48.2 (3.19) ^&^ |
|  | **12** | 82 | 221 | 29.32(10.44) | 34.88(13.20) ^&^ | 42.42 (2.99) | 49.1 ( 3.70) ^&^ |
|  | **13** | 91 | 213 | 33.98(13.44) | 40.84(15.30) ^&^ | 42.12 (3.94) | 49.2(4.29) ^&^ |
|  | **14** | 102 | 237 | 40.07(14.04) | 45.66(15.66) | 42.07 (3.88) | 49.1 (4.68) ^&^ |
|  | **15** | 109 | 192 | 41.65(15.00) | 46.40(16.33) ^&^ | 40.96 (4.56) | 47.7 (5.11) ^&^ |
|  | **16** | 106 | 213 | 42.84(15.76) | 51.87(15.82) ^&^ | 39.42 (4.64) | 48.0 ( 4.81) ^&^ |
|  | **17** | 89 | 192 | 47.63(16.22) | 54.02(14.60) ^&^ | 39.26 (4.70) | 47.4(4.49) ^&^ |
| **Girls** | **9** | 64 | 183 | 19.48(5.52) | 18.91(5.80) | 45.06 (1.85) | 49.2 ( 1.61) ^&^ |
|  | **10** | 86 | 166 | 20.07(5.50) | 21.67(8.09) ^&^ | 43.22 (1.73) | 48.4 ( 2.42) ^&^ |
|  | **11** | 87 | 167 | 22.59(7.06) | 24.65(8.55) | 42.33 (2.37) | 47.4 (2.72) ^&^ |
|  | **12** | 84 | 197 | 22.35(7.23) | 28.63(10.13) ^&^ | 40.53 (2.32) | 47.1 (3.03) ^&^ |
|  | **13** | 89 | 252 | 23.90(8.31) | 34.14(11.42) ^&^ | 39.10 (2.83) | 47.2 ( 3.61) ^&^ |
|  | **14** | 93 | 214 | 24.33(8.32) | 32.78(10.96) ^&^ | 37.54 (2.85) | 45.3 (3.55) ^&^ |
|  | **15** | 85 | 179 | 22.14(7.57) | 30.05(10.05) ^&^ | 35.01 (2.83) | 42.7 (3.45) ^&^ |
|  | **16** | 96 | 219 | 21.64(8.21) | 28.72(9.06) ^&^ | 33.16 (3.10) | 40.7 (3.23) ^&^ |
|  | **17** | 78 | 213 | 21.03 (7.08) | 28.22(9.07) ^&^ | 31.06 (3.04) | 39.3 ( 3.18) ^&^ |

N1= sample size of children and adolescents in Tibet; N2= sample size of children and adolescents in Shanghai; M = Mean; SD = Standard Deviation.

^&^P<0.05 versus Tibet group.
